# The influence of a series of five dry cupping treatments on pain and mechanical thresholds in patients with chronic non-specific neck pain - a randomised controlled pilot study

**DOI:** 10.1186/1472-6882-11-63

**Published:** 2011-08-15

**Authors:** Romy Lauche, Holger Cramer, Kyung-Eun Choi, Thomas Rampp, Felix Joyonto Saha, Gustav J Dobos, Frauke Musial

**Affiliations:** 1 Chair of Complementary and Integrative Medicine, Alfried Krupp von Bohlen und Halbach Foundation, University of Duisburg-Essen, Knappschafts-Krankenhaus, Am Deimelsberg 34a, 45276 Essen, Germany; 2 The National Research Center in Complementary and Alternative Medicine (NAFKAM), Department of Community Medicine, Faculty of Health Science, University of Tromsø, Forskningsparken I, Sykehusveien 23, 9037 Tromsø, Norway

## Abstract

**Background:**

In this preliminary trial we investigated the effects of dry cupping, an ancient method for treating pain syndromes, on patients with chronic non-specific neck pain. Sensory mechanical thresholds and the participants' self-reported outcome measures of pain and quality of life were evaluated.

**Methods:**

Fifty patients (50.5 ± 11.9 years) were randomised to a treatment group (TG) or a waiting-list control group (WL). Patients in the TG received a series of 5 cupping treatments over a period of 2 weeks; the control group did not. Self-reported outcome measures before and after the cupping series included the following: Pain at rest (PR) and maximal pain related to movement (PM) on a 100-mm visual analogue scale (VAS), pain diary (PD) data on a 0-10 numeric rating scale (NRS), Neck Disability Index (NDI), and health-related quality of life (SF-36). In addition, the mechanical-detection thresholds (MDT), vibration-detection thresholds (VDT), and pressure-pain thresholds (PPT) were determined at pain-related and control areas.

**Results:**

Patients of the TG had significantly less pain after cupping therapy than patients of the WL group (PR: Δ-22.5 mm, p = 0.00002; PM: Δ-17.8 mm, p = 0.01). Pain diaries (PD) revealed that neck pain decreased gradually in the TG patients and that pain reported by the two groups differed significantly after the fifth cupping session (Δ-1.1, p = 0.001). There were also significant differences in the SF-36 subscales for bodily pain (Δ13.8, p = 0.006) and vitality (Δ10.2, p = 0.006). Group differences in PPT were significant at pain-related and control areas (all p < 0.05), but were not significant for MDT or VDT.

**Conclusions:**

A series of five dry cupping treatments appeared to be effective in relieving chronic non-specific neck pain. Not only subjective measures improved, but also mechanical pain sensitivity differed significantly between the two groups, suggesting that cupping has an influence on functional pain processing.

**Trial registration:**

The trial was registered at clinicaltrials.gov (NCT01289964).

## Background

Neck pain is a very common condition, the average lifetime prevalence being 48.5% [1]. The causes of chronic neck pain are manifold and can include inflammatory diseases, degenerative processes, trauma, space-occupying lesions, or systemic conditions. However, in most patients neck pain is not due to a serious disease, but rather to postural or mechanical factors. It is then commonly referred to as simple or non-specific neck pain [2]. While non-specific neck pain usually resolves within three to six months, it recurs or persists even longer in 14% of patients [3], who are then considered to have chronic neck pain [4].

Although the pathogenesis of non-specific neck pain is not completely understood, it is agreed that physiological and psychological factors such as stress [3], poor mental health [5,6], long hours of work at a desk, an otherwise heavy workload, little exercise, and postural deficits may contribute to mechanical neck pain [7]. Alterations in connective tissues, such as inflammation and fibrosis [8,9], or in muscles, such as impairment of the microcirculation of the trapezius [10,11], may occur, and motor control of the neck musculature may be affected [12]. Moreover, patients with chronic non-specific neck pain commonly show hyperalgesia, i.e., enhanced sensitivity to mechanical pain [13-17], although it is still under discussion whether the hyperalgesia is localised [15,17] or widespread [14]. Hyperalgesia in chronic non-specific neck pain also shows different patterns and seems to rely on different mechanisms than hyperalgesia in acute [14] and traumatic neck pain [17] respectively.

Conventional treatment of non-specific neck pain includes patient education [18] and physical exercises [19,20], primarily as preventive methods. In more acute or severe cases, spinal manipulation, physical therapy [21,22], or medicinal or injection therapies [23] may be applied. However, additional treatment options are needed, especially for patients with more severe pain [24,25] or with low expectations of conventional treatment alone [26].

A complementary treatment option frequently employed for chronic pain conditions is cupping, an ancient medical technique of European, Asian, and Middle Eastern cultures [27,28]. Each of the various cupping techniques utilizes a glass cup to create suction over a painful area. With dry or fire cupping the cups are applied to the intact skin, while with so-called wet or bloody cupping the skin is incised before the cups are applied. Cupping is applied to increase the local circulation of blood and lymph and to relieve painful muscle tension [29]. In clinical practice cupping is regularly observed to bring about pain relief and to increase a patient's general feeling of wellbeing [28,29].

Although cupping was successfully utilised to treat pain and a wide variety of other complaints for thousands of years, it has almost vanished from the therapeutic spectrum of modern medicine, especially in Europe. Nonetheless, interest in cupping has increased during the last decade since preliminary systematic clinical trials have suggested that cupping is effective in managing painful conditions [30-33]. However, a search of the literature in pubmed, medline, and web of science in April 2010 failed to identify an RCT on dry cupping for the treatment of chronic non-specific neck pain.

The aim of this pilot study was to determine whether a series of cupping treatments effectively relieves chronic non-specific neck pain. In addition, mechanical thresholds of the subjects were measured to determine whether cupping has an effect on mechanical hyperalgesia in patients with chronic neck pain.

## Methods

### Patients

The study protocol was approved by the institutional review board of the University Duisburg-Essen Medical Institutions (no.09-3986). Fifty patients were included in the study between July and November 2009. Inclusion criteria were ages between 18 and 75 and neck pain for at least 5 days a week for at least 3 consecutive months with a mean pain intensity of 40 mm on a 100-mm visual analogue scale (VAS). Patients were included only if specific causes for their neck pain had been excluded at some time by an orthopaedist or a neurologist. An additional inclusion criterion was based on the recommendations for different cupping methods [27,28]. Accordingly, patients eligible for dry cupping showed so-called blank myogeloses, which are hyperirritable areas of skeletal muscle associated with small palpable nodules in taut bands of muscle fibres. These myogeloses are usually associated with increased muscle tension and lowered microcirculation in the affected area. Patients with a voluminous gelosis of the dermis, i.e. connective tissue swelling and adhesions, were not included in this trial but referred for wet cupping.

Exclusion criteria were one or more of the following: neck pain caused by trauma or whiplash, inflammatory or malignant disease, congenital malformation of the spine, or neck pain accompanied by radicular symptoms such as radiating pain, paresis, prickling, or tingling. Patients were also excluded if they had had invasive treatments within the last 4 weeks, surgery to the spine within the last year, or had been treated with corticosteroids or opiates. Further exclusion criteria were serious acute or chronic organic disease such as diabetes or cancer, mental disorders, pregnancy, or a haemorrhagic tendency or anticoagulation treatment. Non-steroidal pain medication and physiotherapy were allowed if the treatment regimen had not been altered for 4 weeks before the trial and were continued during the trial. This ensured that statistical evaluation of the effects of cupping treatments was not influenced by alterations in medications or physiotherapy during the study phase.

All patients were recruited by notices printed in their local newspapers. They were screened twice, first in a standardised telephone interview and second in a physical and neurological examination by the study physician during their first appointment. All participants provided informed written consent.

### Study design

**Figure 1:**
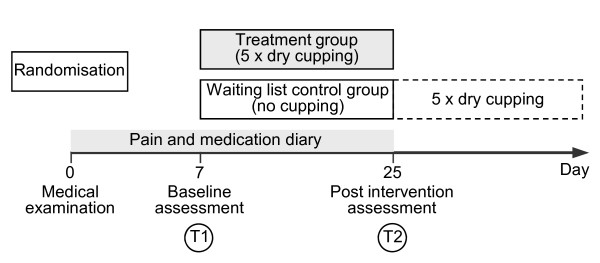
**Study design**.

After being interviewed by telephone, potential participants were invited to be assessed on whether they were eligible for the study. Their informed consent was obtained in written, and they were randomly assigned to either a treatment or a waiting-list control group by means of sequentially numbered, sealed opaque envelopes prepared by the study coordinator, who was neither involved in treatment nor in measurement. Patients were given a pain diary (PD) in which to record their daily medications, changes in symptoms, or other data relevant to the trial. They were then scheduled for measurement and treatment appointments. Figure 1 illustrates the study design.

At baseline assessment (T1) participants filled out questionnaires dealing with their medical history, pain at rest (PR), pain related to movement (PM), Neck Disability Index (NDI), and quality of life (SF-36). Sensory tests were performed that included vibration-detection threshold (VDT), mechanical-detection threshold (MDT), and pressure-pain threshold (PPT). At the end of T1 the treatment group received the first of five dry cupping treatments administered over two weeks, whereas the waiting list control group received no treatment. Participants were assessed a second time 18 days after T1 (T2). They again filled out the questionnaires and underwent sensory testing. The waiting-list control group was offered cupping treatment after they had completed their post-intervention assessment.

### Intervention: dry cupping technique

Cupping was performed by the study physician, who was trained in cupping and regularly performed cupping in a clinical setting. Patients lay prone on a massage couch with their upper torso bared. The study physician used the patient's pain diagram (see Methods: Mechanical sensory and pain thresholds) and physical examination to identify areas of muscle tension and myogeloses, which most commonly occurred in the descending and transverse parts of the trapezius muscle. The cupping procedure was then performed as follows: double-walled glass cups (4-10 glasses with diameters from 25 to 50 mm) were held inverted over an open flame to heat the air inside, after which each glass was placed on an afflicted area. As the air inside the cups cooled, vacuums were created, drawing up the skin within each cup. The glasses were removed after 10 to 20 minutes depending on the colour of the circular so-called cupping marks, which range from slightly rose to dark pink. Cupping marks usually fade away completely after 2-4 days. The procedure was repeated every 3 to 4 days. A total of five cupping treatments was chosen, which on the one hand was considered the minimal number to demonstrate any significant effects of cupping treatment and on the other hand would ensure that the trial could feasibly be carried out.

### Expectation

It is well known that a patient's expectation of the effectiveness of a treatment may influence the outcome of the treatment. Therefore, after the participants in this trial were randomised to their respective groups, they were asked to rate their expectations of the cupping treatments they were to receive on a visual analogue scale from 0 = "not effective at all" to 100 mm = "most effective".

### Outcome measures

#### Pain

Pain at rest (PR) and maximal pain related to movement (PM, provoked pain by neck flexion, neck extension, lateral neck flexion, and neck rotation in either direction) were recorded on a VAS graded from 0 (no pain at all) to 100 mm (worst pain imaginable). For PM the movement direction with the highest pain rating at T1 was chosen for each patient. Baseline and post-intervention pain scores were recorded at T1 and T2. In addition patients kept a pain and medication diary (the PDs utilized a numeric rating scale, or NRS, graded from 0 to 10) from day 0 (7 days prior to T1) until T2.

#### Questionnaires

The Neck Disability Index (NDI) [34] was used at T1 and T2 to assess the patient's perceived disability associated with neck pain. Health-related quality of life was quantified by the German version of the SF-36 [35,36]. The SF-36 provides a detailed health profile on the basis of eight health dimensions as well as sum scores for physical and mental health. The standard version (4-week time frame) was used for baseline assessment at T1 and the acute recall version (1-week time frame) at T2. The latter version was used at T2 because it was considered more sensitive to recent changes in health status [37].

#### General Health outcome

Within the SF-36 the General Health outcome was recorded on a 5-point Likert scale that ranged from "My health is much better than before treatment" to "My health is much worse than before treatment".

#### Mechanical sensory and pain thresholds

Sensory testing included mechanical-detection threshold (MDT), pressure-pain threshold (PPT), and vibration-detection threshold (VDT) and was conducted in four areas: two pain-related areas and two control areas. Control areas were located on the right hand and foot. The pain-related areas were individually determined for each patient. First, the patient was given a diagram of the body on which she/he was told to mark the most painful spot in his neck and shoulder region. This spot, defined as that patient's site of maximal pain (Pain-Maximum), was verified by physical examination. A second point, defined as Pain-Adjacent, was chosen adjacent to the painful area, i.e., the patient did not report pain in that area. Again physical examination was used to confirm the patient's information. Both locations were marked on the pain diagram so that they could be precisely located for the repeat measurements at T2. All sensory measurements were determined and calculated according to the Quantitative Sensory Testing (QST) standardised protocol developed by Rolke et al. [38,39] to ensure inter-study comparability. The QST sensory tests indicate whether sensitivity in certain modalities is heightened or diminished. Retest- and inter-observer-reliability with standardized QST have proven satisfactory [40].

The mechanical-detection threshold (MDT) was quantified using a set of 17 von Frey filaments (Aesthesiometer, SOMEDIC, Sweden) at a patient's Pain-Maximum and Pain-Adjacent points, on the back of their right hand, and on the dorsum of their right foot according to the QST protocol [38]. Upon bending, the Aesthesiometer exerts forces between 0.26 and 1080 mN. With a starting force of 16 mN, the next lower hair was applied until the subject no longer felt the stimulus. Then the next stronger hair was applied until the subject could feel the stimulus again. Using the method of limits, the log-transformed geometrical mean of five ascending and descending series was taken as the individual's MDT.

The pressure-pain threshold (PPT) was measured by a pressure algometer (Algometer, SOMEDIC, Sweden) at Pain-Maximum and Pain-Adjacent and the patient's right thenar eminence and right instep. It exerts forces of up to 2000 kPa when used with a probe area of 1 cm^2^. The pressure pain threshold was measured in 3 ramps of increasing pressure intensities of ca. 50 kPa/s until the subject signalled the first feeling of pain in addition to the pressure sensation. The log-transformed arithmetic mean of these three series was taken as the individual's PPT [38].

The vibration-detection threshold (VDT) was quantified by a Rydel Seiffer tuning fork (64 Hz, 8/8 scale). It was placed over a bony prominence, e.g., on a spinous process, the styloid process of the ulna, or the lateral malleolus and left there until the subject could not feel the vibration anymore. The arithmetic mean of three series was taken as the individual's VDT [38].

#### Reliability of threshold measurements

To evaluate the reliability of the sensory threshold measurements, the retest reliabilities were determined at the control areas in the control group participants (WL, N = 24). Correlation coefficients were r = 0.57 for MDT Hand (p = 0.004), r = 0.53 for MDT Foot (p = 0.008), r = 0.73 for PPT Hand (p = 0.000004), r = 0.74 for PPT Foot (p = 0.00004), r = 0.6 for VDT Hand (p = 0.002) and r = 0.77 for VDT Foot (p = 0.00001).

The average correlation coefficients was r = 0.65 which indicates sufficient reliability.

#### Side effects

All participants were asked to report any side effects during the treatment period. The questionnaires relating to T2 also included an open question about relevant experiences and side effects.

### Statistical Analyses

The treatment and waiting list control groups were compared using chi-square analysis for discrete data and independent t-tests for continuous data on demographic, pain history, and pre-treatment variables to ensure the comparability at baseline. For each outcome measure except the pain diary we compared the results of the intervention by analysis of covariance (ANCOVA) taking the post-treatment measurement (T2) as a dependent and group as a between-subject factor. Respective baseline values of the outcome (T1) and expectancy served as covariates. The intention-to-treat principle was applied in this study. Missing data of the TG participant who dropped out during treatment was filled in with the subject's last observation.

Pain diaries were analysed by means of a repeated measurement ANCOVA. The data were condensed as follows: 1) pain ratings of the week before T1 were arithmetically averaged and served as baseline; 2) since the gaps of time between interventions differed among the subjects, pain ratings between two sessions or between session 5 and T2 were averaged, resulting in five post-intervention measures. For the WL control group the number of days between T1 and T2 was divided by the number of treatments in the TG, i.e., pain ratings were averaged every 3.5 days to ensure comparability between the groups. Within the ANCOVA model the group variable served as the between-subject factor; the post-intervention measures served as the dependent factors; and baseline and expectancy served as the covariates. Medications recorded in the daily diaries were converted into relative number of days under medication.

The General Health outcome was analysed by means of the Mann-Whitney U test.

The level of statistical significance was adjusted using the Bonferroni-Holm correction within each test. An alpha of 0.05 was chosen for all other analyses.

## Results

### CONSORT Flowchart

After the first telephone screening, 75 patients were invited for further evaluation. 50 of them fulfilled the study criteria and agreed to participate in the study.

**Figure 2:**
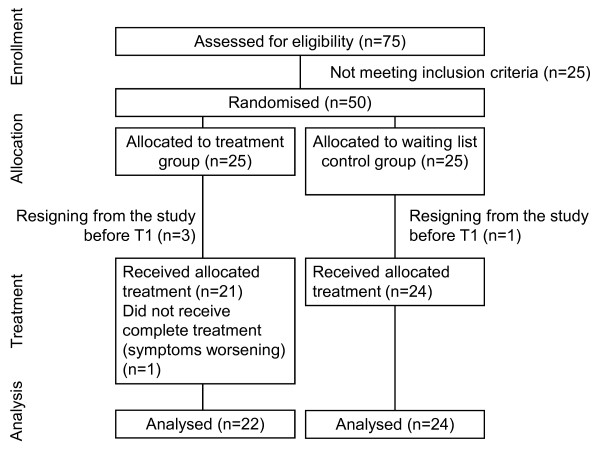
**Consort flow chart of recruitment and trial conditions**.

Three participants in the treatment group and one in the waiting list control group resigned for personal reasons; no data were collected from these participants. One participant in the treatment group discontinued treatment because of worsening symptoms. In this case last-observation data were carried forward. Final analyses were conducted on 22 participants in the treatment group and on 24 participants in the waiting list control group. Figure 2 shows a flow chart of patient recruitment.

### Sample characteristics

Study participants had a long history of neck pain, on average of 7.2 ± 6.9 years duration. The majority reported that their pain was permanent and that they had no pain-free intervals (93%). The two groups were comparable in age, sex ratio, and clinical characteristics; see table 1.

**Table 1:** Baseline Demographic and Clinical Characteristics of Trial Groups

SOCIODEMOGRAPHIC AND CLINICAL CHARACTERISTICS	TREATMENT GROUP (N = 22) MEAN ± SD	WAITING LIST CONTROL GROUP (N = 24) MEAN ± SD	P
Age (years)	48.6 ± 11.2	53.0 ± 11.4	0.20
Sex (F/M)	15/7	20/4	0.27
BMI (kg/m^2^)	24.9 ± 4.0	24.1 ± 3.1	0.47
Pain at rest (PR)	45.5 ± 20.9	42.3 ± 18.0	0.58
Duration of neck pain (years)	6.3 ± 6.1	8.0 ± 7.6	0.41
Expected effectiveness of cupping therapy (VAS from 0 = not effective at all to 100 = highly effective)	82.8 ± 13.6	72.4 ± 21.3	0.06

Pre- and post-intervention scores and estimated differences are presented in table 2 and described in detail below.

**Table 2:** Outcomes of pain measures and questionnaires at T1, T2 and estimated group differences at T2

	T1		T2		ESTIMATED DIFFERENCE AT T2	ANCOVA		
	**TREATMENT GROUP** **(N = 22)** **(MEAN ± SD)**	**WAITING LIST CONTROL GROUP** **(N = 24)** **(MEAN ± SD)**	**TREATMENT GROUP** **(N = 22)** **(MEAN ± SD)**	**WAITING LIST CONTROL GROUP** **(N = 24)** **(MEAN ± SD)**	DIFF TREATMENT GROUP VS. WAITING LIST CONTROL GROUP* (95% CI)	**DF**	**F**	**P**

Pain at rest (PR)	45.5 ± 20.9	42.3 ± 18.0	26.1 ± 22.7	47.1 ± 19.8	-22.5 (-31.9 to -13.1)	45	23.4	0.00002
Pain at movement (PM)	62.0 ± 31.2	58.4 ± 22.2	29.0 ± 26.9	45.5 ± 25.3	-17.8 (-31.3 to -4.6)	45	8.2	0.01
Neck Disability Index (NDI)	27.5 ± 12.1	29.1 ± 10.5	21.1 ± 11.2	29.2 ± 8.4	-6.3 (-10.2 to -2.4)	45	10.8	0.002
SF-36 Physical functioning	80.3 ± 11.3	76.7 ± 11.4	83.0 ± 13.6	79.4 ± 10.2	2.5 (-3.6 to 8.5)	45	0.7	0.41
SF-36 Role-physical	55.7 ± 39.3	37.5 ± 31.3	78.4 ± 31.1	57.3 ± 35.7	16.1 (-4.9 to 37.0)	45	2.4	0.13
SF-36 Bodily pain	46.9 ± 14.7	40.9 ± 8.4	60.3 ± 16.7	43.8 ± 15.0	13.8 (4.2 to 23.4)	45	8.4	0.006
SF-36 General Health Perception	65.9 ± 21.1	58.1 ± 18.5	65.5 ± 23.5	56.8 ± 16.8	3.7 (-4.7 to 12.0)	45	0.8	0.38
SF-36 Vitality	55.0 ± 17.4	46.2 ± 18.3	63.9 ± 16.4	46.7 ± 16.7	10.2 (3.0 to 17.3)	45	8.3	0.006
SF-36 Social function	79.5 ± 25.5	65.6 ± 26.9	91.4 ± 19.0	70.3 ± 27.5	11.4 (0.6 to 22.2)	45	4.5	0.04
SF-36 Role emotional	71.2 ± 38.9	58.3 ± 38.4	86.4 ± 30.3	68.1 ± 39.9	12.9 (-8.1 to 34.0)	45	1.5	0.11
SF-36 Mental Health	49.2 ± 11.0	43.9 ± 12.1	79.8 ± 13.7	64.3 ± 18.5	8.5 (1.9 to 15.1)	45	6.8	0.1
SF-36 **Physical Component Score**	42.8 ± 5.7	40.2 ± 5.1	45.7 ± 6.4	42.3 ± 6.1	3.0 (-0.8 to 6.8)	45	2.6	0.12
SF-36 **Mental Component Score**	49.2 ± 11.0	43.9 ± 12.1	54.2 ± 8.9	45.0 ± 13.1	5.0 (-0.2 to 10.1)	45	3.8	0.06

* Group differences and P values from an ANCOVA model with 2 groups, baseline values and expectancy as covariates

### Pain

After cupping, the two groups differed significantly for pain at rest (PR); the estimated group difference was -22.5 mm (95% CI -31.9 to -13.1, p = 0.00002) on the VAS. The same effect was found for maximal pain related to movement (PM), with an estimated group difference of -18.8 mm (95% CI -32.0 to -5.6, p = 0.01).

**Figure 3:**
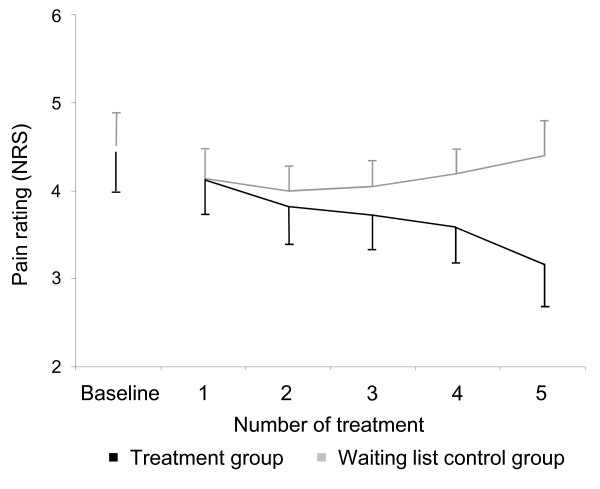
**Pain ratings decrease in the treatment group (pain diary, NRS, Mean ± SEM)**. Pain ratings of the TG were averaged between two cupping sessions. For the WL group we divided the number of days between T1 and T2 by the number of treatments in the TG. This resulted in pain ratings averaged every 3.5 days.

Analyses of the pain diaries (PD) by means of repeated measurement ANCOVA revealed a significant Time × Group interaction (F = 3.5, df = 4.80, ε = 0.03, p = 0.026). Post hoc analyses showed that the groups differed significantly after the 5th cupping treatment (Δ-1.1, 95% CI 0.5 to 1.8, p = 0.001). With single comparisons within the TG, pain ratings after the 1st and the 5th cupping treatments also differed significantly (Δ-0.9, 95% CI -1.5 to -0.4, p = 0.002). The course of the pain diary data is shown in Figure 3.

The medication diary data could not be analysed by means of ANCOVA because more than 86.9% of the participants in the TG had not taken any pain medications in the week before T1. Similarly, this data was not considered eligible for other statistical analyses because of the large percentage of non-medicated patients.

### Questionnaires

The Neck Disability Index (NDI) differed significantly between the two groups at T2, the estimated difference being -6.3% (95%CI -10.2 to -2.4, p = 0.002). The Physical or Mental Component Scores (SF-36) did not differ significantly between the two groups, although the Mental Component Score showed a strong trend (p = 0.06). Subscale analysis of the SF-36 revealed significant group differences in bodily pain (Δ13.8, 95% CI 4.2 to 23.4, p = 0.006) and vitality (Δ10.2, 95% CI 3.0 to 17.3, p = 0.006), indicating less pain and greater vitality after cupping. A significant group difference was found with the General Health Outcome evaluation (Mann Whitney U Test, Mean Rank TG: 18.1; WL: 28.5, U = 144.0, p = 0.002). In particular, 9 of 22 TG participants reported that their health had improved at least somewhat between T1 and T2 (much better N = 3, somewhat better N = 6), whereas no WL participants reported improvement. The majority of WL patients rated their health about the same as before (N = 18); a minority considered it somewhat worse (N = 6). Interestingly, 2 TG participants felt worse at T2 than at T1 (somewhat worse N = 1, much worse N = 1), although these same participants reported less pain (PR) at T2.

### Mechanical sensory and pain thresholds

**Figure 4:**
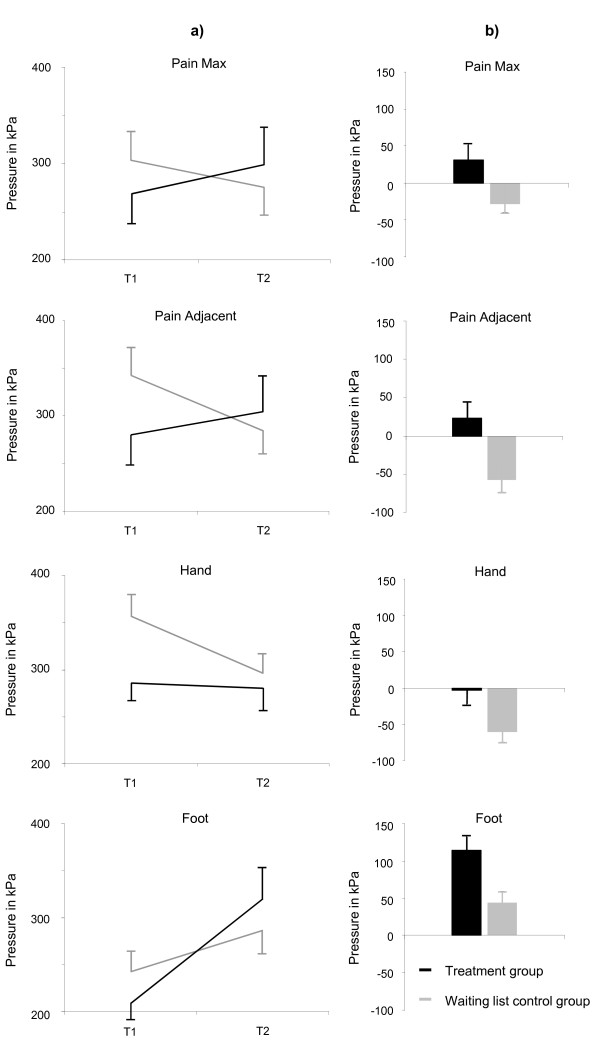
**Course of pressure-pain thresholds over time (a) and differences in pressure-pain thresholds (T2-T1) (b) at pain-related and control areas (Mean ± SEM)**. Please note: Raw data instead of log-transformed data were used for illustration purposes.

The two groups showed significant differences for PPT, but not for MDT or VDT, see table 3. Significant group differences in PPT were found at pain-related areas and at control areas (Pain-Maximum: Δ 0.08, 95% CI 0.01 to 0.16, p = 0.026; Pain-Adjacent: Δ 0.11, 95% CI 0.05 to 0.17, p = 0.001; Hand: Δ 0.07, 95% CI 0.01 to 0.14, p = 0.003; Foot: Δ 0.12, 95% CI 0.04 to 0.20, p = 0.004). Figure 4 shows the course of pressure-pain thresholds in all areas. Whereas the PPTs at pain-related areas and on the right hands were stable or increased in TG patients, they decreased in these areas in the WL group. PPTs on the right feet increased in both groups.

**Table 3:** Mechanical detection and pain thresholds at T1, T2 and estimated group differences at T2 (Mean ± SD)

		T1		T2		ESTIMATED DIFFERENCE AT T2	ANCOVA		
		**TREATMENT GROUP** **(N = 22)** **(MEAN ± SD)**	**WAITING LIST CONTROL GROUP** **(N = 24)** **(MEAN ± SD)**	**TREATMENT GROUP** **(N = 22)** **(MEAN ± SD)**	**WAITING LIST CONTROL GROUP** **(N = 24)** **(MEAN ± SD)**	DIFF TREATMENT GROUP VS. WAITING LIST CONTROL GROUP* (95% CI)	**DF**	**F**	**P**

MDT lg(mN)	Pain Maximum	0.58 ± 0.42	0.29 ± 0.44	0.51 ± 0.35	0.38 ± 0.41	0.007 (-0.21 to 0.22)	1/42	0.00	0.95
	Pain Adjacent	0.34 ± 0.41	0.25 ± 0.31	0.32 ± 0.44	0.25 ± 0.37	0.003 (-0.21 to 0.21)	1/42	0.00	0.98
	Hand	0.08 ± 0.40	0.19 ± 0.35	0.07 ± 0.39	0.16 ± 0.44	0.04 (-0.16 to 0.24)	1/42	0.18	0.67
	Foot	0.42 ± 0.41	0.55 ± 0.31	0.41 ± 0.37	0.58 ± 0.27	-0.06 (-0.20 to 0.09)	1/42	0.03	0.44
VDT x/8	Pain Maximum	6.45 ± 0.96	5.93 ± 1.06	6.80 ± 1.11	6.28 ± 0.96	0.15 (-0.29 to 0.59)	1/42	0.47	0.50
	Pain Adjacent	6.17 ± 0.98	5.43 ± 1.02	6.69 ± 1.18	5.82 ± 1.05	0.29 (-0.23 to 0.82)	1/42	1.26	0.27
	Hand	7.53 ± 0.56	7.26 ± 0.80	7.39 ± 0,76	7.10 ± 0.68	0.14 (-0.22 to 0.49)	1/42	0.61	0.44
	Foot	6.03 ± 1.17	5.78 ± 1.02	6.17 ± 1.22	5.75 ± 1.01	0.35 (-0.06 to 0.76)	1/42	3.05	0.09
PPT lg(kPa)	Pain Maximum	2.36 ± 0.25	2.43 ± 0.24	2.41 ± 0.26	2.39 ± 0.20	0.08 (0.01 to 0.16)	1/42	5.35	0.026
	Pain Adjacent	2.40 ± 0.21	2.50 ± 0.19	2.44 ± 0.21	2.43 ± 0.15	0.11 (0.05 to 0.17)	1/42	13.23	0.001
	Hand	2.43 ± 0.16	2.53 ± 0.15	2.44 ± 0.16	2.44 ± 0.16	0.07 (0.01 to 0.14)	1/42	4.78	0.034
	Foot	2.29 ± 0.18	2.35 ± 0.18	2.48 ± 0.19	2.41 ± 0.21	0.12 (0.04 to 0.20)	1/42	9.13	0.004

MDT = mechanical-detection threshold

PPT = pressure-pain threshold

VDT = vibration-detection threshold

An ANCOVA model with 2 groups, baseline values and expectancy as covariates revealed significant group differences in PPT at all areas.

## Discussion

### Principal findings

Patients of the TG reported significant symptomatic improvement after cupping treatments: Pain at rest (PR), maximal pain related to movement (PM), the Neck Disability Index (NDI), and bodily pain (SF-36) decreased after repeated cupping. Pain ratings from the pain diaries (PD) decreased significantly after the fifth treatments. The effects of medication on treatment outcomes could not be evaluated since the majority of patients did not use any medication at all. According to the quality of life questionnaires (SF-36), cupping treatments also significantly decreased bodily pain and improved vitality. In addition, the mental component score showed a strong trend, although non-significant.

Cupping also showed an effect on one of the non-subjective parameters, the pressure-pain threshold (PPT), which is thought to reflect the functional status of (altered) pain perception. Pressure pain thresholds at pain-related areas and on the hand increased or remained stable over time in the TG, whereas patients of the WL control group became sensitised at those areas. PPTs on the foot increased in both groups, but the effect was twice as large in the TG as in the WL control group.

### Interpretation of the findings

In this study various pain measures such as pain at rest (PR), pain related to movement (PM), pain diary (PD) data, and bodily pain (SF-36) differed significantly between the TG and the WL after cupping. Thus, repeated dry cupping appears to be effective in treating chronic non-specific neck pain. Since changes in the VAS and the NDI were also strongly correlated in the TG (r = 0.69, N = 22), pain relief would appear to be associated with reduced impairment. However, fewer than 10 points of improvement of the NDI are not clinically significant by definition, so that these changes cannot be considered clinically significant for the TG as a whole.

Interestingly, the pain diary ratings indicate that the effects of cupping are likely cumulative. That is, cupping is more effective as a serial treatment than as a one-time treatment. This conforms to standard clinical practise, in which cupping treatments are usually applied as a series. Cupping is administered as an adjuvant to standard or alternative therapy in the majority of cases. Cupping may act alone or enhance other therapies by stretching muscle and connective tissue and thereby decreasing TGF-β1 and collagen synthesis [41], which are known to trigger fibrosis and connective tissue stiffness [8]. It may further enhance microcirculation, cellular metabolism, and regeneration.

Furthermore, vitality as reflected by the SF-36 changed significantly. Since patients with lower scores on bodily pain and vitality are more likely to use CAM [42], the observed changes may be due to decreased affective-emotional distress. Stress is known to increase neck pain intensity by increasing muscle tension, and 47.8% of our study cohort reported that stress exacerbates their symptoms. That is, cupping may relieve stress and pain perception not only by specific effects but also by unspecific effects or means [43] such as expectation, conditioning, or regulation of the autonomic nervous system. When patients in this trial were asked to rate how relaxed they were during cupping on a 100-mm VAS from 0 = "not relaxed at all" to 100 mm = "very relaxed", they scored on average 91.2 ± 8.9 mm (Mean ± SD).

### Pressure pain thresholds

Low pressure-pain thresholds are commonly found in various pain conditions; they indicate that pain perception has been altered by sensitization at one or more levels of pain processing. Various therapies such as massage [44] or manipulation [45] have been shown to increase pressure-pain thresholds.

The pressure-pain thresholds found in this trial presented a complex pattern. Here three points are noteworthy: 1) while thresholds at pain-related areas and on the hand followed similar patterns, those on the feet showed a different pattern. Sensitization (i.e., decreased thresholds) did not occur with repeated measurements at the foot in either the TG or the WL group. Possibly pain processing is altered to different degrees in patients with chronic non-specific neck pain. Hyperalgesia associated with chronic non-specific neck pain is localised, unlike that associated with neck pain due to whiplash [15,17]. However, pressure-pain thresholds may decrease not only within the area of neck pain, but also within the trigeminal region whiplash [15]. Since painful area and pain adjacent are close together by definition and painful area and hand are segmental, this might explain the diverse effects at the pain-related areas, the hand, and the foot.

2) The effects at pain-related areas and the hand are not only due to significant changes within TG but also to sensitization in WL. Although the reliability of PPTs has rarely been investigated and has not been established, it seems that if PPTs are measured on consecutive days they decrease [45], whereas if measured over longer time intervals they remain steady or even increase [46]. Short-term observations of PPTs in patients with chronic non-specific neck pain indicated that they decrease after a single day, as in healthy controls [47]. The recovery of pressure-pain thresholds in patients with chronic neck pain and probable altered pain processing might be disturbed due to a) continuing nociceptive input and b) dysfunctional regeneration of muscle and deep tissue. Consequently the decrease in pressure-pain thresholds in the WL group is likely to persist. Since alterations in functional pain processing are probably segmental, the foot may show an inverse effect. There, thresholds increased even in the WL group.

3) In the TG the PPTs remained steady or increased, probably because of the effects of the cupping treatments. Interestingly these effects were apparently present at pain-related areas, the hand, and even the foot, where both groups became less sensitive to pressure pain, with the effect more pronounced in the TG than in the WL group. This systemic effect may be the result of immunological responses. Blood that has extravasated during cupping triggers a resorption response [28] that is closely linked to hemoxygenase-1 (HO-1) gene expression [48,49], which in turn is associated with cytoprotective and antinociceptive effects [49-51]. Or it may be related to stimulation itself, which has been shown to induce changes of the hormonal and the emotional status [52]. This interpretation is speculative and the hypothesis needs further elucidation. Other causes, for example unspecific treatment effects, could not yet be ruled out because suitable sham devices are presently not available [43].

### Patients' evaluation

Patients were asked how they had experienced the cupping treatments to help determine whether cupping had unspecific effects. We asked the participants of the TG if they experienced changes of any kind. Most of these patients reported that they had less neck pain, that their neck and shoulder muscles had become softer and more relaxed (11×), and that their neck and shoulder regions had become more mobile (4×). As side effects they reported a tingling sensation in their hands and arms (1×), strain/pain at the treated area (2) or in their general neck region (1×), slight headache (1×), tiredness (1×), a shivering attack (1×), blurred vision (1×), and improved nasal breathing (1×). Whether the latter are directly related to the treatments is not clear, but none of the "side effects" persisted longer than 4 hours and no permanent side effects were reported. One patient did discontinue treatment because their symptoms temporarily worsened.

On a visual analogue scale ranging from 0 = "no benefit at all" to 100 mm = "maximum possible benefit" patients rated their benefit 60.4 ± 27.0 mm (Mean ± SD) on average. Of the 22 participants, 19 would consider continuing cupping therapy and 21 of 22 would recommend cupping therapy to their family and friends.

### Limitations of the study

Results of the study might be limited due to the small sample size and the choice of the passive control group. A sham control group was not included because a reliable sham cupping intervention is presently not available. Sham cupping that utilizes adhesives to keep the cups in place in our experience can usually be recognized by the patients, even those inexperienced with cupping. Besides, changes in pain scores in waiting list control groups and placebo groups in trials of conventional treatments for chronic non-specific neck pain are usually comparable [53]. Another problem in such a trial is that experimental blinding of the assessor is impracticable because the cupping marks are often visible and may persist for several days. The early randomization at day 0 also might have affected baseline values and treatment outcomes. Nevertheless, baseline values were comparable between the groups and all treatment outcomes were corrected for expectation by means of covariance analyses. Allowing both groups to use non-steroidal pain medication and physiotherapy may have influenced the outcomes. But since the vast majority of patients did not use either of these therapies their influence was considered insignificant.

### Strengths of the study

Despite the limitations of the study, the pain reduction (VAS) of approximately 44.8% (95% CI -59.1 to -30.6) observed in the treatment group is within the range of clinical relevance which is defined as a minimal clinical change of 2 points on the NRS or 30% pain reduction [54]. The observed effect size for pain at rest (PR) was d = 1.4, which is considered a large effect size. Moreover cupping also showed an effect on pressure-pain thresholds, which are less likely to be influenced by patient bias than simple pain ratings.

## Conclusions

A series of five dry cupping sessions appear to be safe and effective in treating chronic non-specific neck pain. The procedure was well accepted by the patients. Further randomized controlled studies are warranted to confirm these results and to compare the effectiveness of cupping treatments with placebo treatments or standard care. In addition, further investigations on the physiology of pain processing and mechanisms of action of cupping are needed.

## Competing interests

All authors disclosed any commercial association that might create a conflict of interest in connection with the submitted manuscript. There is especially no competing financial interest for any of the authors.

## Authors' contributions

RL was responsible for the conception and design of the study, the implementation of sensory testing, recruitment, acquisition, analysis and interpretation of data and drafting and revising the manuscript. HC was responsible for the recruitment, acquisition, analysis and interpretation of the data and revising the manuscript. KC was responsible for the conception and design of the study, for analysis and interpretation of the data and revising the manuscript. TR and FS were responsible for the study design and the patients' examination and intervention. GD was responsible for the conception and design of the study and critically revising the manuscript. FM was responsible for the conception and design of the study and introduced neurophysiologic methods for sensory threshold measurements, participated in the analysis and interpretation of the data and revised the manuscript. All authors read and approved the final manuscript.

## Pre-publication history

The pre-publication history for this paper can be accessed here:

http://www.biomedcentral.com/1472-6882/11/63/prepub
